# *N*,*N*′-Bis(4-hy­droxy­phen­yl)pyridine-2,6-dicarboxamide di­methyl­formamide monosolvate

**DOI:** 10.1107/S1600536813013810

**Published:** 2013-05-31

**Authors:** Ghulam Waris, Humaira Masood Siddiqi, Ulrich Flörke, Rizwan Hussain, M. Saeed Butt

**Affiliations:** a Department of Chemistry, Quaid-I-Azam University, Islamabad 45320, Pakistan; b Universität Paderborn, Warburgerstrasse 100, D-33098 Paderborn, Germany; c NESCOM, PO Box 2216 Islamabad, Pakistan

## Abstract

The mol­ecular structure of the pyridine derivative, C_19_H_15_N_3_O_4_·C_3_H_7_NO, shows almost planar geometry with dihedral angles of 6.9 (1) and 13.4 (1)° between the pyridine ring and the two benzene rings. This conformation is stabilized by two intra­molecular N—H⋯N(pyridine) bonds. In the crystal, strong O—H⋯O(carboxamide) and N—H⋯O(hy­droxy­phen­yl) hydrogen bonds link the mol­ecules, forming a three-dimensional structure. The di­methyl­formamide solvent mol­ecules are not involved in the hydrogen bonding. The structure shows pseudosymmetry, but refinement in the space group *Pbcn* leads to significantly worse results and a disordered di­methyl­formamide mol­ecule.

## Related literature

For applications of aromatic polyamides, see: Hamciuc *et al.*, (2001 ▶); Yang *et al.* (1998 ▶); Diakoumakos & Mikroyannidis (1994 ▶); Ebadi & Mehdipour-Ataei (2010 ▶). For the structure of a related Co-complex, see: Ali *et al.* (2012 ▶).
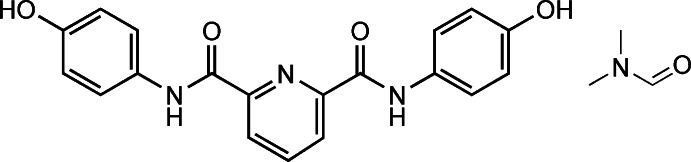


## Experimental

### 

#### Crystal data

C_19_H_15_N_3_O_4_·C_3_H_7_NO
*M* *_r_* = 422.44
Orthorhombic, 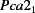

*a* = 16.8124 (12) Å
*b* = 10.9545 (8) Å
*c* = 10.9331 (7) Å
*V* = 2013.6 (2) Å^3^
*Z* = 4
Mo *K*α radiation
μ = 0.10 mm^−1^
*T* = 130 K
0.47 × 0.41 × 0.39 mm

#### Data collection

Bruker SMART APEX diffractometer
Absorption correction: multi-scan (*SADABS*; Sheldrick, 2004 ▶) *T*_min_ = 0.954, *T*_max_ = 0.962
18386 measured reflections
2536 independent reflections
2434 reflections with *I* > 2σ(*I*)
*R*_int_ = 0.022

#### Refinement

*R*[*F* ^2^ > 2σ(*F* ^2^)] = 0.036
*wR*(*F* ^2^) = 0.099
*S* = 1.03
2536 reflections
284 parameters
1 restraint
H-atom parameters constrained
Δρ_max_ = 0.33 e Å^−3^
Δρ_min_ = −0.32 e Å^−3^

### 

Data collection: *SMART* (Bruker, 2002 ▶); cell refinement: *SAINT* (Bruker, 2002 ▶); data reduction: *SAINT*; program(s) used to solve structure: *SHELXTL* (Sheldrick, 2008 ▶); program(s) used to refine structure: *SHELXTL*; molecular graphics: *SHELXTL*; software used to prepare material for publication: *SHELXTL* and local programs.

## Supplementary Material

Crystal structure: contains datablock(s) I, global. DOI: 10.1107/S1600536813013810/bt6908sup1.cif

Structure factors: contains datablock(s) I. DOI: 10.1107/S1600536813013810/bt6908Isup2.hkl

Supplementary material file. DOI: 10.1107/S1600536813013810/bt6908Isup3.cml

Additional supplementary materials:  crystallographic information; 3D view; checkCIF report

## Figures and Tables

**Table 1:** Hydrogen-bond geometry (Å, °)

*D*—H⋯*A*	*D*—H	H⋯*A*	*D*⋯*A*	*D*—H⋯*A*
N2—H2*B*⋯N1	0.88	2.20	2.661 (2)	113
N3—H3*A*⋯N1	0.88	2.22	2.675 (2)	112
O2—H2⋯O1^i^	0.84	1.92	2.7572 (19)	179
O4—H4⋯O3^ii^	0.84	1.91	2.7464 (19)	172
N2—H2*B*⋯O2^iii^	0.88	2.44	3.125 (2)	135
N3—H3*A*⋯O4^iv^	0.88	2.41	3.043 (2)	130

Symmetry codes: (i) 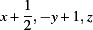; (ii) 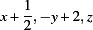; (iii) 
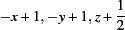
; (iv) 
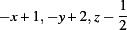
.

**Fig. 1.**
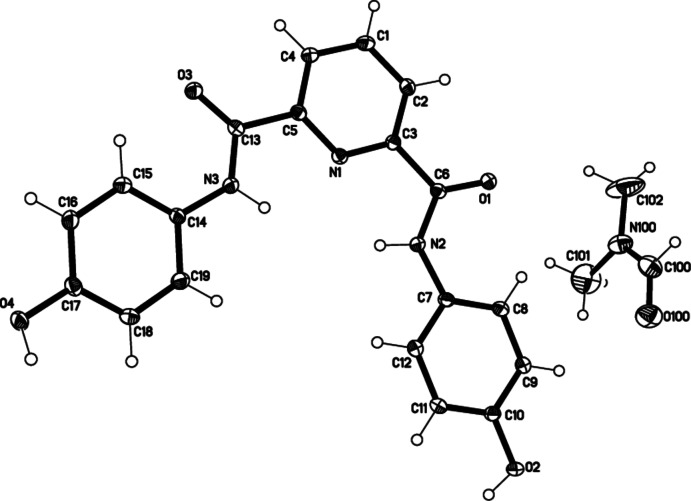
Molecular structure of the title compound with anisotropic displacement parameters drawn at the 50% probability level.

**Fig. 2.**
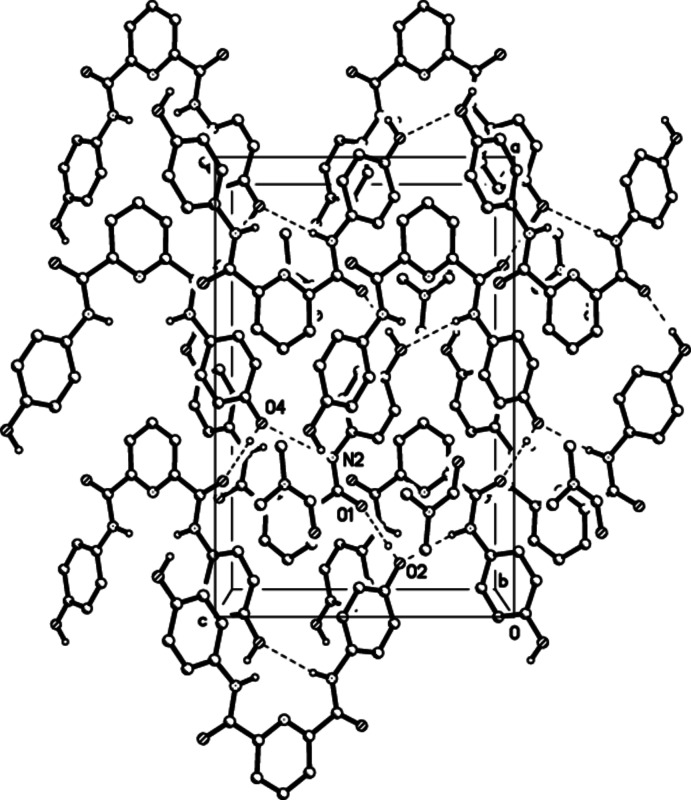
Crystal packing viewed along b axis with intermolecular hydrogen bonds as dotted lines. H-atoms not involved are omitted.

## supplementary crystallographic information

### Comment

Aromatic polyamides' application in novel technologies has grown rapidly due to their usage as a beneficial alternative for metals and other goods (Yang, *et al.*, 1998, Hamciuc *et al.*, 2001). The company of amide linkages makes them strong applicant for semi-permeable membrane as they are hydrophilic polymers and water absorbent. In addition their high tech artificial fibre usage in the production of defensive attire of protective firemen, soldiers, race car drivers and gas filteration makes them prominent among others (Ebadi, *et al.*, 2010, Diakoumakos *et al.*, 1994). As part of our ongoing research in solubility of aromatic poly(amide-imide)s by structural modification, we are reporting a pyridine-based monomer having inbuilt amide functionality. It enhances the solubility of resulting poly(amid-imide)s without deteriorating the inherent properties of the polymer.

### Experimental

This preparation was carried out by using reagent grade quality chemicals without their further purification. In a 100 ml, three necked, round bottomed flask, equipped with a condenser, a nitrogen gas inlet tube, a thermometer and a magnetic stirrer, 0.02mole (2.18 g m) of 4-hydroxyaniline in 30 mL of dry tetrahydrofuran(THF) stirred at 273–278 K for 30 minutes and 0.01 mol (2.04 g m) of pyridine -2,6-dicarbonyl dichloride in 35 mL of THF was added dropwise by dropping funnel and stirring was continued for further 1 h under same conditions. The temperature of reaction mixture was then raised to 308–313 K and stirring was continued for 30 minutes. The flask content was cooled to room temperature, poured into water and let it for 24 h. Resulting purplish precipitates were filtered, washed with hot water and 5% NaOH solution. Finally, product was washed with hot water and methanol, dried under vacuum at 80°C. The crude product was recrystallized from tetrahydrofuran and dimethylformamide(4:1).

### Refinement

Hydrogen atoms were clearly identified in difference syntheses, refined at idealized positions riding on the carbon, nitrogen or oxygen atoms with C–H 0.95–0.98, N–H 0.88, O–H 0.84 Å and with isotropic displacement parameters *U*_iso_(H) = 1.2*U*_eq_(C/N) or 1.5*U*_eq_(–CH_3_ and –OH H atoms). All CH_3_ and OH hydrogen atoms were allowed to rotate but not to tip.

The title compound crystallizes in the non-centrosymmetric space group *Pca*2_1_; however, in the absence of significant anomalous scattering effects, the Flack parameter is essentially meaningless. Accordingly, Friedel pairs were merged.

Refinement in space group *Pbcn* with both molecules on special positions gives 359 systematic absence violations (in *Pca*2_1_ only 3, all with I less than 3σ), most of them with I>3σ(I) and significantly worse refinement parameters *R*1 = 0.107, *wR*2 = 0.284, *S* = 1.24.

### Crystal data

**Table:**

C_19_H_15_N_3_O_4_·C_3_H_7_NO	*D*_x_ = 1.393 Mg m^−^^3^
*M**_r_* = 422.44	Mo *K*α radiation, λ = 0.71073 Å
Orthorhombic, *P**c**a*2_1_	Cell parameters from 8531 reflections
*a* = 16.8124 (12) Å	θ = 2.2–28.3°
*b* = 10.9545 (8) Å	µ = 0.10 mm^−^^1^
*c* = 10.9331 (7) Å	*T* = 130 K
*V* = 2013.6 (2) Å^3^	Prism, pale-pink
*Z* = 4	0.47 × 0.41 × 0.39 mm
*F*(000) = 888	

### Data collection

**Table:**

Bruker SMART APEX diffractometer	2536 independent reflections
Radiation source: sealed tube	2434 reflections with *I* > 2σ(*I*)
Graphite monochromator	*R*_int_ = 0.022
φ and ω scans	θ_max_ = 27.9°, θ_min_ = 1.9°
Absorption correction: multi-scan (*SADABS*; Sheldrick, 2004)	*h* = −22→22
*T*_min_ = 0.954, *T*_max_ = 0.962	*k* = −14→14
18386 measured reflections	*l* = −14→14

### Refinement

**Table:**

Refinement on *F*^2^	Primary atom site location: structure-invariant direct methods
Least-squares matrix: full	Secondary atom site location: difference Fourier map
*R*[*F*^2^ > 2σ(*F*^2^)] = 0.036	Hydrogen site location: difference Fourier map
*wR*(*F*^2^) = 0.099	H-atom parameters constrained
*S* = 1.03	*w* = 1/[σ^2^(*F*_o_^2^) + (0.0683*P*)^2^ + 0.4544*P*] where *P* = (*F*_o_^2^ + 2*F*_c_^2^)/3
2536 reflections	(Δ/σ)_max_ < 0.001
284 parameters	Δρ_max_ = 0.33 e Å^−^^3^
1 restraint	Δρ_min_ = −0.32 e Å^−^^3^

### Special details

**Table:**

Geometry. All e.s.d.'s (except the e.s.d. in the dihedral angle between two l.s. planes) are estimated using the full covariance matrix. The cell e.s.d.'s are taken into account individually in the estimation of e.s.d.'s in distances, angles and torsion angles; correlations between e.s.d.'s in cell parameters are only used when they are defined by crystal symmetry. An approximate (isotropic) treatment of cell e.s.d.'s is used for estimating e.s.d.'s involving l.s. planes.
Refinement. Refinement of *F*^2^ against ALL reflections. The weighted *R*-factor *wR* and goodness of fit *S* are based on *F*^2^, conventional *R*-factors *R* are based on *F*, with *F* set to zero for negative *F*^2^. The threshold expression of *F*^2^ > σ(*F*^2^) is used only for calculating *R*-factors(gt) *etc*. and is not relevant to the choice of reflections for refinement. *R*-factors based on *F*^2^ are statistically about twice as large as those based on *F*, and *R*- factors based on ALL data will be even larger.

### Fractional atomic coordinates and isotropic or equivalent isotropic displacement parameters (Å^2^)

**Table:**

	*x*	*y*	*z*	*U*_iso_*/*U*_eq_	
O1	0.22086 (7)	0.57263 (12)	0.50884 (13)	0.0191 (3)	
O2	0.58528 (7)	0.39649 (13)	0.37035 (15)	0.0215 (3)	
H2	0.6268	0.4049	0.4122	0.032*	
O3	0.22824 (7)	0.93446 (12)	1.04434 (13)	0.0202 (3)	
O4	0.59295 (8)	1.09650 (14)	1.18167 (15)	0.0242 (3)	
H4	0.6353	1.0812	1.1440	0.036*	
N1	0.25192 (7)	0.75080 (13)	0.77701 (18)	0.0152 (2)	
N2	0.33363 (8)	0.62143 (14)	0.61383 (17)	0.0183 (3)	
H2B	0.3500	0.6615	0.6789	0.022*	
N3	0.33916 (8)	0.87656 (14)	0.93824 (16)	0.0180 (3)	
H3A	0.3544	0.8341	0.8741	0.022*	
C1	0.08639 (9)	0.7555 (2)	0.7806 (2)	0.0224 (3)	
H1A	0.0299	0.7566	0.7819	0.027*	
C2	0.12663 (10)	0.69108 (18)	0.69062 (19)	0.0189 (4)	
H2A	0.0984	0.6479	0.6291	0.023*	
C3	0.20912 (10)	0.69128 (16)	0.69270 (17)	0.0153 (3)	
C4	0.12932 (10)	0.81813 (18)	0.8684 (2)	0.0193 (4)	
H4A	0.1031	0.8635	0.9305	0.023*	
C5	0.21208 (10)	0.81302 (15)	0.86329 (17)	0.0152 (3)	
C6	0.25491 (11)	0.62261 (14)	0.59558 (19)	0.0152 (3)	
C7	0.39446 (10)	0.56544 (16)	0.54420 (19)	0.0165 (4)	
C8	0.38133 (10)	0.48237 (17)	0.44972 (19)	0.0188 (4)	
H8A	0.3286	0.4630	0.4253	0.023*	
C9	0.44580 (10)	0.42785 (18)	0.3914 (2)	0.0192 (4)	
H9A	0.4369	0.3711	0.3271	0.023*	
C10	0.52331 (10)	0.45623 (16)	0.42674 (19)	0.0170 (4)	
C11	0.53648 (10)	0.54289 (17)	0.51766 (19)	0.0181 (4)	
H11A	0.5892	0.5652	0.5395	0.022*	
C12	0.47240 (10)	0.59636 (17)	0.57604 (19)	0.0180 (4)	
H12A	0.4815	0.6549	0.6386	0.022*	
C13	0.26050 (10)	0.88030 (15)	0.95796 (19)	0.0157 (4)	
C14	0.40125 (10)	0.93161 (17)	1.00651 (19)	0.0165 (4)	
C15	0.38915 (11)	1.02561 (18)	1.0904 (2)	0.0202 (4)	
H15A	0.3369	1.0536	1.1075	0.024*	
C16	0.45424 (12)	1.07814 (19)	1.1488 (2)	0.0214 (4)	
H16A	0.4460	1.1425	1.2057	0.026*	
C17	0.53075 (10)	1.03808 (18)	1.12533 (19)	0.0186 (4)	
C18	0.54283 (11)	0.94174 (19)	1.0435 (2)	0.0204 (4)	
H18A	0.5950	0.9121	1.0286	0.024*	
C19	0.47829 (10)	0.88971 (17)	0.9842 (2)	0.0198 (4)	
H19A	0.4866	0.8249	0.9278	0.024*	
O100	0.33017 (10)	0.30965 (16)	0.17286 (19)	0.0424 (4)	
N100	0.21891 (10)	0.2555 (2)	0.2759 (3)	0.0341 (4)	
C100	0.25928 (14)	0.31580 (19)	0.1894 (3)	0.0342 (5)	
H100	0.2297	0.3678	0.1368	0.041*	
C101	0.2617 (2)	0.1731 (3)	0.3556 (4)	0.0661 (10)	
H101	0.2472	0.0887	0.3361	0.099*	
H102	0.2479	0.1907	0.4409	0.099*	
H103	0.3191	0.1841	0.3438	0.099*	
C102	0.13354 (17)	0.2626 (4)	0.2881 (5)	0.0805 (13)	
H104	0.1127	0.3235	0.2308	0.121*	
H105	0.1199	0.2864	0.3719	0.121*	
H106	0.1101	0.1828	0.2699	0.121*	

### Atomic displacement parameters (Å^2^)

**Table:**

	*U*^11^	*U*^22^	*U*^33^	*U*^12^	*U*^13^	*U*^23^
O1	0.0161 (5)	0.0232 (6)	0.0180 (7)	0.0000 (5)	−0.0017 (5)	−0.0037 (6)
O2	0.0134 (6)	0.0297 (7)	0.0215 (7)	0.0051 (5)	0.0001 (6)	−0.0053 (6)
O3	0.0157 (6)	0.0250 (6)	0.0200 (7)	−0.0009 (5)	0.0019 (6)	−0.0036 (6)
O4	0.0147 (6)	0.0375 (8)	0.0204 (8)	−0.0070 (5)	0.0008 (6)	−0.0074 (7)
N1	0.0134 (5)	0.0171 (5)	0.0150 (6)	−0.0018 (8)	0.0006 (8)	0.0015 (4)
N2	0.0137 (7)	0.0237 (7)	0.0175 (8)	0.0013 (5)	−0.0002 (6)	−0.0057 (7)
N3	0.0144 (7)	0.0224 (7)	0.0171 (8)	−0.0006 (5)	0.0008 (7)	−0.0045 (7)
C1	0.0121 (6)	0.0301 (8)	0.0252 (8)	0.0008 (8)	0.0013 (9)	−0.0048 (7)
C2	0.0161 (8)	0.0226 (8)	0.0182 (10)	−0.0017 (6)	−0.0024 (7)	−0.0035 (8)
C3	0.0148 (7)	0.0169 (7)	0.0142 (9)	0.0005 (6)	0.0004 (7)	0.0006 (7)
C4	0.0145 (7)	0.0239 (9)	0.0195 (9)	0.0023 (6)	0.0010 (7)	−0.0025 (8)
C5	0.0148 (7)	0.0157 (7)	0.0151 (9)	−0.0001 (6)	−0.0006 (7)	0.0018 (7)
C6	0.0141 (7)	0.0159 (7)	0.0156 (9)	−0.0003 (6)	−0.0004 (7)	0.0023 (7)
C7	0.0131 (7)	0.0198 (8)	0.0167 (9)	0.0020 (6)	0.0002 (7)	0.0014 (8)
C8	0.0133 (7)	0.0232 (8)	0.0201 (10)	−0.0011 (6)	−0.0011 (7)	−0.0028 (8)
C9	0.0176 (8)	0.0226 (9)	0.0173 (8)	0.0008 (7)	−0.0021 (7)	−0.0025 (7)
C10	0.0146 (7)	0.0197 (8)	0.0167 (9)	0.0023 (6)	0.0005 (7)	0.0011 (8)
C11	0.0136 (7)	0.0213 (8)	0.0193 (9)	0.0008 (6)	−0.0022 (7)	−0.0001 (7)
C12	0.0167 (8)	0.0202 (8)	0.0172 (10)	−0.0002 (6)	−0.0015 (7)	−0.0023 (7)
C13	0.0145 (8)	0.0159 (7)	0.0167 (9)	−0.0007 (6)	−0.0015 (7)	0.0018 (7)
C14	0.0126 (7)	0.0205 (8)	0.0163 (9)	−0.0016 (6)	−0.0007 (7)	−0.0013 (7)
C15	0.0143 (7)	0.0245 (8)	0.0217 (9)	0.0006 (7)	0.0007 (7)	−0.0041 (8)
C16	0.0193 (8)	0.0250 (9)	0.0198 (10)	−0.0030 (7)	0.0002 (8)	−0.0063 (8)
C17	0.0161 (8)	0.0251 (9)	0.0148 (9)	−0.0052 (6)	−0.0002 (7)	0.0014 (8)
C18	0.0138 (7)	0.0245 (8)	0.0228 (9)	−0.0003 (6)	0.0013 (8)	−0.0009 (8)
C19	0.0168 (8)	0.0205 (8)	0.0220 (10)	−0.0002 (6)	0.0019 (7)	−0.0031 (7)
O100	0.0323 (8)	0.0469 (10)	0.0478 (11)	−0.0081 (7)	0.0065 (8)	0.0085 (9)
N100	0.0273 (8)	0.0307 (8)	0.0442 (10)	−0.0010 (8)	0.0072 (11)	−0.0014 (7)
C100	0.0339 (11)	0.0272 (9)	0.0415 (14)	0.0009 (9)	−0.0054 (11)	0.0063 (11)
C101	0.069 (2)	0.076 (2)	0.053 (2)	−0.0001 (18)	0.0106 (18)	0.0381 (19)
C102	0.0282 (13)	0.089 (3)	0.124 (4)	−0.0040 (15)	0.026 (2)	−0.037 (3)

### Geometric parameters (Å, º)

**Table:**

O1—C6	1.236 (2)	C9—C10	1.394 (2)
O2—C10	1.376 (2)	C9—H9A	0.9500
O2—H2	0.8400	C10—C11	1.392 (3)
O3—C13	1.240 (2)	C11—C12	1.382 (3)
O4—C17	1.372 (2)	C11—H11A	0.9500
O4—H4	0.8400	C12—H12A	0.9500
N1—C3	1.339 (2)	C14—C15	1.394 (3)
N1—C5	1.343 (2)	C14—C19	1.396 (2)
N2—C6	1.338 (2)	C15—C16	1.392 (3)
N2—C7	1.415 (2)	C15—H15A	0.9500
N2—H2B	0.8800	C16—C17	1.383 (3)
N3—C13	1.341 (2)	C16—H16A	0.9500
N3—C14	1.418 (2)	C17—C18	1.398 (3)
N3—H3A	0.8800	C18—C19	1.387 (3)
C1—C4	1.383 (3)	C18—H18A	0.9500
C1—C2	1.387 (3)	C19—H19A	0.9500
C1—H1A	0.9500	O100—C100	1.207 (3)
C2—C3	1.387 (2)	N100—C100	1.339 (3)
C2—H2A	0.9500	N100—C102	1.444 (3)
C3—C6	1.512 (3)	N100—C101	1.446 (4)
C4—C5	1.394 (2)	C100—H100	0.9500
C4—H4A	0.9500	C101—H101	0.9800
C5—C13	1.509 (2)	C101—H102	0.9800
C7—C8	1.394 (3)	C101—H103	0.9800
C7—C12	1.397 (2)	C102—H104	0.9800
C8—C9	1.392 (3)	C102—H105	0.9800
C8—H8A	0.9500	C102—H106	0.9800
			
C10—O2—H2	109.5	C11—C12—C7	120.87 (18)
C17—O4—H4	109.5	C11—C12—H12A	119.6
C3—N1—C5	117.56 (13)	C7—C12—H12A	119.6
C6—N2—C7	129.70 (17)	O3—C13—N3	124.65 (17)
C6—N2—H2B	115.2	O3—C13—C5	121.33 (15)
C7—N2—H2B	115.2	N3—C13—C5	114.01 (17)
C13—N3—C14	128.95 (17)	C15—C14—C19	119.57 (17)
C13—N3—H3A	115.5	C15—C14—N3	123.58 (16)
C14—N3—H3A	115.5	C19—C14—N3	116.83 (17)
C4—C1—C2	119.34 (15)	C16—C15—C14	119.53 (17)
C4—C1—H1A	120.3	C16—C15—H15A	120.2
C2—C1—H1A	120.3	C14—C15—H15A	120.2
C1—C2—C3	118.38 (18)	C17—C16—C15	120.99 (18)
C1—C2—H2A	120.8	C17—C16—H16A	119.5
C3—C2—H2A	120.8	C15—C16—H16A	119.5
N1—C3—C2	123.33 (17)	O4—C17—C16	118.52 (18)
N1—C3—C6	116.88 (15)	O4—C17—C18	121.91 (17)
C2—C3—C6	119.79 (17)	C16—C17—C18	119.55 (17)
C1—C4—C5	118.27 (19)	C19—C18—C17	119.72 (17)
C1—C4—H4A	120.9	C19—C18—H18A	120.1
C5—C4—H4A	120.9	C17—C18—H18A	120.1
N1—C5—C4	123.12 (18)	C18—C19—C14	120.62 (18)
N1—C5—C13	117.43 (15)	C18—C19—H19A	119.7
C4—C5—C13	119.45 (17)	C14—C19—H19A	119.7
O1—C6—N2	124.63 (18)	C100—N100—C102	122.9 (3)
O1—C6—C3	121.56 (16)	C100—N100—C101	118.78 (19)
N2—C6—C3	113.81 (17)	C102—N100—C101	118.2 (3)
C8—C7—C12	119.42 (17)	O100—C100—N100	125.4 (2)
C8—C7—N2	124.55 (16)	O100—C100—H100	117.3
C12—C7—N2	116.02 (17)	N100—C100—H100	117.3
C9—C8—C7	119.75 (16)	N100—C101—H101	109.5
C9—C8—H8A	120.1	N100—C101—H102	109.5
C7—C8—H8A	120.1	H101—C101—H102	109.5
C8—C9—C10	120.31 (18)	N100—C101—H103	109.5
C8—C9—H9A	119.8	H101—C101—H103	109.5
C10—C9—H9A	119.8	H102—C101—H103	109.5
O2—C10—C11	121.57 (16)	N100—C102—H104	109.5
O2—C10—C9	118.51 (17)	N100—C102—H105	109.5
C11—C10—C9	119.92 (17)	H104—C102—H105	109.5
C12—C11—C10	119.64 (17)	N100—C102—H106	109.5
C12—C11—H11A	120.2	H104—C102—H106	109.5
C10—C11—H11A	120.2	H105—C102—H106	109.5
			
C4—C1—C2—C3	0.5 (4)	C9—C10—C11—C12	2.8 (3)
C5—N1—C3—C2	0.0 (3)	C10—C11—C12—C7	−0.6 (3)
C5—N1—C3—C6	179.48 (14)	C8—C7—C12—C11	−2.0 (3)
C1—C2—C3—N1	−0.2 (3)	N2—C7—C12—C11	177.10 (18)
C1—C2—C3—C6	−179.65 (18)	C14—N3—C13—O3	−0.8 (3)
C2—C1—C4—C5	−0.5 (4)	C14—N3—C13—C5	178.39 (17)
C3—N1—C5—C4	−0.1 (3)	N1—C5—C13—O3	−176.12 (17)
C3—N1—C5—C13	−179.83 (14)	C4—C5—C13—O3	4.1 (3)
C1—C4—C5—N1	0.4 (3)	N1—C5—C13—N3	4.7 (2)
C1—C4—C5—C13	−179.91 (18)	C4—C5—C13—N3	−175.07 (18)
C7—N2—C6—O1	−0.7 (3)	C13—N3—C14—C15	−16.9 (3)
C7—N2—C6—C3	178.91 (17)	C13—N3—C14—C19	164.9 (2)
N1—C3—C6—O1	−174.51 (16)	C19—C14—C15—C16	1.3 (3)
C2—C3—C6—O1	5.0 (3)	N3—C14—C15—C16	−176.87 (19)
N1—C3—C6—N2	5.9 (2)	C14—C15—C16—C17	−0.3 (3)
C2—C3—C6—N2	−174.64 (18)	C15—C16—C17—O4	177.3 (2)
C6—N2—C7—C8	−10.6 (3)	C15—C16—C17—C18	−1.3 (3)
C6—N2—C7—C12	170.39 (19)	O4—C17—C18—C19	−176.8 (2)
C12—C7—C8—C9	2.4 (3)	C16—C17—C18—C19	1.8 (3)
N2—C7—C8—C9	−176.64 (18)	C17—C18—C19—C14	−0.8 (3)
C7—C8—C9—C10	−0.2 (3)	C15—C14—C19—C18	−0.8 (3)
C8—C9—C10—O2	177.25 (18)	N3—C14—C19—C18	177.52 (19)
C8—C9—C10—C11	−2.4 (3)	C102—N100—C100—O100	−177.4 (3)
O2—C10—C11—C12	−176.86 (19)	C101—N100—C100—O100	−1.6 (4)

### Hydrogen-bond geometry (Å, º)

**Table:**

*D*—H···*A*	*D*—H	H···*A*	*D*···*A*	*D*—H···*A*
N2—H2*B*···N1	0.88	2.20	2.661 (2)	113
N3—H3*A*···N1	0.88	2.22	2.675 (2)	112
O2—H2···O1^i^	0.84	1.92	2.7572 (19)	179
O4—H4···O3^ii^	0.84	1.91	2.7464 (19)	172
N2—H2*B*···O2^iii^	0.88	2.44	3.125 (2)	135
N3—H3*A*···O4^iv^	0.88	2.41	3.043 (2)	130

Symmetry codes: (i) *x*+1/2, −*y*+1, *z*; (ii) *x*+1/2, −*y*+2, *z*; (iii) −*x*+1, −*y*+1, *z*+1/2; (iv) −*x*+1, −*y*+2, *z*−1/2.
